# Berlin Medical Society

**Published:** 1888-08

**Authors:** 


### THE BERLIN MEDICAL SOCIETY.

### THE TREATMENT OF GALL STONES.

Dr. Rosenberg related his experiences in the treatment of gall-stones, by olive oil, as recently recommended from America. A patient suffering from marked biliary colic, that had continued for five years, and had proved refractory in spite of all treatment, including a "Trinkkur" in Carlsbad, and in whom the pain was very acute, was given a dose of 100 grammes of olive oil (about 3 ozs.) at night. In the morning there were found in the stool three concretions, one the size of a grain of linseed, the other two smaller. Some days later a second dose was given of 180 grammes, and in the stool that followed 180 concretions were found, varying in size from a pin head to a hazel nut, and after a third dose 243 stones. The patient's troubles soon after this began to diminish, but they did not entirely disappear. She took in all, in five doses, 820 grms., of olive oil, and 629 stones were counted in the evacuations that followed. The gall bladder, which before was the size of the fist and projected beyond the edge of the liver, diminished so far that it could be no longer palpated. It was assumed that the oil did not act by exciting peristalsis of the intestines, but by passing directly into the gall bladder and softening the contents. Vomiting only once resulted from the administration.

After Dr. Sonnenburg had shown a case of osteo-sarcoma of the femur,

Dr. Casper introduced the subject of the Radical Cure of Prostatic Enlargement and Tumors by Electrolysis. He remarked at the commencement that the treatment that had hitherto received the sanction of the most distinguished authorities, Thompson, Guyon, Dittel, and Socin, was merely palliative, although such treatment had been acknowledged to be powerless in arresting the progress of the disease. Attempts had been made in various directions, such as extirpation or crushing of the excrescence from the urethra, injection of iodine or carbolic acid from the rectum, excision by the galvano-cautery from the urethra, or diminution of the mass by ignipuncture from the rectum. Again, the gland had been removed wholly or in part by the high operation. Although more recently such distinguished surgeons as Thompson, von Langenbeck, and Benno-Schmidt, had expressed the opinion that in suitable cases prostatectomy should be done in the future, the speaker was of opinion that a less formidable procedure should first be given a trial. Such a procedure he believed he had found in electrolysis. Since this method of treating tumors was first introduced in 1864 by Nélaton, it had been successfully employed by others. As the decomposed constituents of the prostatic tumor could not make their exit externally, but must of necessity be removed by absorption into the tissues, he had performed experiments on animals with a view of ascertaining whether such absorption entailed any dangers as regarded embolism or otherwise. He treated four rabbits by passing the negative electrode, with cutaneous portion insulated, into a mass of muscle, and continued a current of 15 to 20 milliamperes for 10 to 15 minutes. No sign of embolism followed. The testicles of dogs were treated in the same way, and after the lapse of a year the animals were perfectly healthy, except that the testicles were reduced to a small residuum. He then proceeded to carry out the treatment on men. The patient was placed on the side, the bowel washed out with a 1-1000th solution of sublimate, and the external electrode, 480 ctm. square, placed on the abdomen. The internal electrode, properly guarded, was then passed to the spot selected for puncture, the mucous membrane smoothed out and inserted in the direction desired. The current was then turned on, gradually increasing from 10 to 25 milliamperes; after the lapse of five minutes the needle was partially withdrawn, and passed in another direction, and then after another five minutes in another, so that the sitting altogether lasted fifteen minutes. The pain was quite "nominal"; the current caused a slight burning and pricking in the penis. The application was repeated at intervals, twenty or more times. Retention of urine and bladder irritation were simultaneously treated by the classical methods. Four cases were thus treated, and of this number two were much improved both subjectively and objectively. In the third case a slight improvement took place; the fourth was no better, and in consequence of the insulating material of the electrode getting worn a vesico-rectal fistula resulted, which the author, however, claimed to have been of life-saving service to the patient. In the two cases that were improved the prostate distinctly shriveled up, the residuum of urine after micturition was reduced from 140 or 300 cctm., to 20 or 40 cctm., and the urine only required to be passed every three or four hours, so that a fair night's sleep became once more possible. Pain during micturition also ceased, and the general condition became much better. *Résumé:* The operation was free from danger *quoad oitam*. The danger of a fistulous opening remaining might be excluded. In cases not too far advanced improvement might be expected. Four groups of cases afforded no prospect of improvement by electrolysis. First, those in which the prostate was enlarged rather in thickness than in length or breadth, in which a constant residuum of urine was left after micturition. Second, cases in which immoderate dilatation of the bladder had long existed, so that it did not properly contract even when reduction had been affected in the size of the prostate. Third, cases of so-called concentrically hypertrophied bladder, where the hypertrophy of the bladder walls was so great that the viscus could scarcely act as a container of urine at all. Fourth, those cases in which the hypertrophy of the prostate was so small that it could not be detected from the rectum. Here the case was one rather of a growth at the neck of the bladder, that closed the opening like a valve, and could not well be reached from the rectum. Finally, those cases were to be excluded in which the symptoms, though similar to those of enlargement of the prostate, were due to other causes. In the majority of cases of prostatic enlargement he was convinced that electrolytic treatment would reduce the size of the organ and obviate the dreaded consequences entailed by the condition.

Dr. FURSTENHEIM agreed with the speaker that nothing could be expected of electrolysis in cases in which retention of urine was always present after micturition. Where there was incomplete paralysis, a certain atony and insufficiency of bladder power, the case was different, benefit might reasonably be expected. He recommended the use of the speculum for introducing the needle, so that the "disagreeable accident" of puncture of the numerous hæmorrhoidal veins present in old people, might be avoided.

Dr. VON BERGMANN believed more in digital exploration than in determining the amount of hypertrophy by the measure of the residuum of urine. Patients that came into the clinic with prostatic enlargement, and had their bladders washed out twice a day always improved in that respect. It did not depend entirely on the hypertrophy, but on the other disturbances that were relieved by treatment.

In reply, Dr. CASPER said that there could be no doubt as to the diminution in size of the prostate by electrolysis; this could always be attained. He believed that the residuum of urine left in the bladder was the only reliable measure that gave a constant result. The dangers of the electrolytic operation were not very great, as in the four cases the rectum was punctured more than forty times, and all the four patients were rejoicing in good health.

